# A 10-year retrospective study of methicillin-resistant *Staphylococcus aureus* from burn wound infection in southeast China from 2013 to 2022

**DOI:** 10.3389/fmicb.2023.1301744

**Published:** 2023-12-01

**Authors:** Feifei Gu, Weiping He, Dedong Zhu, Peilang Yang, Jingyong Sun, Lizhong Han

**Affiliations:** ^1^Department of Laboratory Medicine, Ruijin Hospital, Shanghai Jiao Tong University School of Medicine, Shanghai, China; ^2^Department of Clinical Microbiology, Ruijin Hospital, Shanghai Jiao Tong University School of Medicine, Shanghai, China; ^3^Department of Clinical Laboratory, Huaihe Hospital of Henan University, Kaifeng, Henan, China; ^4^Department of Burn, Ruijin Hospital, Shanghai Jiao Tong University School of Medicine, Shanghai, China

**Keywords:** methicillin-resistant *Staphylococcus aureus*, *Staphylococcus aureus*, burn wound infection, burns, epidemiology

## Abstract

**Background:**

Methicillin-resistant *Staphylococcus aureus* (MRSA) is one of the most commonly encountered pathogens among burn patients incurring substantial morbidity and mortality. To investigate the epidemiology and features of MRSA in burn wound infections, we conducted a 10-year retrospective study on MRSA isolated from burn patients with burn wound infections from southeast China from 2013 to 2022.

**Methods:**

One hundred MRSA isolates (10 isolates each year) from burn wound infection among burn patients from 2013 to 2022 were randomly selected and enrolled. In addition to the clinical data of the 100 burn patients, MRSA isolates were characterized by antimicrobial susceptibility testing, detection of toxin genes, and molecular typing.

**Results:**

The median time from the onset of burns and admission to MRSA detected was 13 and 5 days, respectively. No MRSA isolate was found resistant to quinupristin/dalfopristin, linezolid, and vancomycin. Toxin gene *seg* was found most frequently (90%) followed by *sea* (70%) and *eta* (64%). CC8 (74%), ST239 (70%), and SCC*mec* III (72%) were the most common CC, ST, and SCC*mec* types, respectively.

**Conclusion:**

ST239-III (70%) was the predominant clone found in MRSA from burn wound infection among burn patients in southeast China. ST239-III was less found from 2018 to 2022. A higher diversity of MRSA clones was observed in these recent 5 years than that from 2013 to 2017.

## Introduction

Millions of people get burned across the world every year. Burns are one of the most devastating injuries leading to high mortality and morbidity (Mater et al., 2020; Savetamal, 2021). Secondary to the loss of the skin barrier and suppression of the immune system, burn patients are more likely to develop invasive infections, leading to serious complications and even death. As the fourth most common type of trauma across the world, the World Health Organization (WHO) has estimated that ~11 million burn patients require medical attention and result in ~265,000 deaths each year (Mater et al., 2020). In China, approximately 5,000–10,000 people per million suffer burn injuries, and 10% of them require medical intervention (Chen et al., 2018).

*Staphylococcus aureus* is a serious public health concern and an important clinical pathogen that can cause a variety of infections in the skin and soft tissue, respiratory tract, bloodstream, and catheter (Jiang et al., 2018; Mahmoudi et al., 2019). *S. aureus*, particularly methicillin-resistant *S. aureus* (MRSA), remains a leading cause of gram-positive burn wound infections worldwide and has been one of the major contributors to increase the morbidity and mortality rates (Branski et al., 2009). Researchers analyzed 29 articles on multidrug-resistant bacteria outbreaks in burn units, and they found that one of the most frequent bacteria was MRSA (Girerd-Genessay et al., 2016). MRSA burn wound infection can potentially cause a fatal sequence of burn wound sepsis, invasive infection, septicemia, multiple organ failure, and even death (Kalligeros et al., 2019). Moreover, MRSA has been the second most common pathogen only to *Pseudomonas aeruginosa* among burn patients with bacteremia as reported (Kalligeros et al., 2019).

To learn more about the characteristics of MRSA from burn wound infections, we carried out a 10-year retrospective study of MRSA isolated from burn patients with burn wound infections from 2013 to 2022 in Ruijin Hospital in Shanghai, hosting mostly burn patients from southern China. In addition, we would also explore the dynamics of the epidemiology of MRSA in burn wound infections over these 10 years.

## Materials and methods

### Study design

This study was carried out in Ruijin Hospital, Shanghai Jiao Tong University School of Medicine, a tertiary teaching hospital in Shanghai, with more than 3,000 beds serving patients from all over China. Department of Burn is one of the best burn units in China providing medical care to burn victims across the country, mainly from southeast China.

Clinical diagnosis of burn wound infections relies on the appearance of the wound, laboratory abnormalities, and wound cultures (D'Abbondanza and Shahrokhi, 2021). Changes in the appearance of the burn wound, including conversion of partial-thickness to full-thickness injury, loss of previously viable skin grafts, rapid cellulitis expansion of healthy tissue surrounding the injury, rapid eschar separation, or tissue necrosis, may indicate an acute infection. This typically manifests as some combination of pain, purulence, edema, malodor, or discoloration of the skin graft or donor site. Systemic alterations and laboratory abnormalities may help to diagnose burn wound infections. Leukocytosis (white blood cell count of >10,000 cells/mm^3^) and/or multiple protein biomarkers including acute-phase reactants (C-reactive protein/erythrocyte sedimentation rate and sedimentation rate), anticoagulant factors, cytokines, and tissue injury biomarkers (serum lactate) may or may not be associated with burn wound infections. Surface wound swabs and cultures may help identify the predominant pathogen and can be used as surveillance if there is any clinical concern about changes in the burn wound (Church et al., 2006; D'Abbondanza and Shahrokhi, 2021; Ladhani et al., 2021). Diagnosis of burn wound infections can be complicated and requires comprehensive consideration. The burn patients with burn wound infections enrolled in this study were diagnosed by MRSA-positive cultures of wound swabs combined with clinical signs and symptoms and/or laboratory results mentioned above.

This study was approved by the Ethics Committee of Ruijin Hospital, Shanghai Jiao Tong University School of Medicine, and the review committee removed the requirement of informed consent for this retrospective study, which just focused on bacteria and did not involve patient interventions.

### MRSA isolates

From January 2013 to December 2022, 410 non-repetitive MRSA isolates were collected from cultures of wound swabs from burn patients with burn wound infections in Ruijin Hospital, and 100 MRSA isolates were randomly selected and enrolled (10 isolates each year) using the random number generation function of Microsoft Office Excel 2016 (Microsoft Corporation, Redmond, WA, USA). The initial species were identified by MALDI-TOF mass spectrometry (bioMérieux, Marcy-l'Étoile, France).

### Antimicrobial susceptibility testing

The broth microdilution method was used for antimicrobial susceptibility testing in accordance with the guidelines of the Clinical and Laboratory Standards Institute issued in 2023 (CLSI 2023) (Institute CaLS., 2023). Antibiotics selected and tested are presented in **Table 2**. *S. aureus* ATCC29213 strain was included as a control strain for the antimicrobial susceptibility testing.

### Detection of toxin genes

Thirteen significant toxin genes were detected by polymerase chain reaction (PCR) containing *lukS/F-PV* (encoding Panton-Valentine leukocidin), *tst* (encoding toxic shock syndrome toxin 1), *eta* and *etb* (encoding exfoliative toxin A and B), and *sea*-*see* and *seg*-*sej* (encoding staphylococcal enterotoxins SEA-SEE and SEG-SEJ) as described previously (Gu et al., 2020).

### Molecular typing

Multilocus sequence typing (MLST), *spa* typing, SCC*mec* typing, and *agr* typing were performed by PCR and/or sequencing as described previously (Gu et al., 2016, 2020). *mecA* detection was performed by PCR to confirm the MRSA strains.

### Statistical analysis

The t-test, chi-square test, or Fisher's exact test was performed for statistical analysis as appropriate, and a two-sided *P* < 0.05 was considered statistically significant. In this study, statistical analysis was performed using the SAS 8.2 software package (SAS Institute Inc., Cary, NC, USA).

## Results

### Clinical data

From January 2013 to December 2022, the median age of burn patients with MRSA burn wound infection was 37 years (range: 10 months−89 years; interquartile range: 15–54 years), and the sex distribution (male/female) was 75/25% (*P* < 0.0001). The median time from the onset of burns to receiving medical attention was 1 day (range: 0.5 h−90 days); the median time from admission to detection of MRSA was 5 days (range: 1–54 days), and the median time from the onset of burns to MRSA detected was 13 days (range: 1–98 days). Burn by direct exposure to flame accounted for 45%, and scalding accounted for 50%. Furthermore, the incidence of burn by direct exposure to flame was higher in 2013–2017 (*P* = 0.0090), and scalding was higher in 2018–2022 (*P* = 0.0164). More information about the burn patients with MRSA burn wound infection is presented in Table 1.

**Table 1:** Clinical data of burn patients with MRSA burn wound infection from 2013 to 2022.

	**Rate (%)**			
	**Total (*****n*** = **100)**	**2013–2017 (*****n*** = **50)**	**2018–2022 (*****n*** = **50)**	***P*** **-value**
**Demographic characteristic**			
Age, median (range)	37 (10 months−89 years)	33 (10 months−89 years)	41 (10 months−85 year)	0.1711
Male sex	75	37	38	0.8174
**Diagnosis and comorbidities**			
Diabetes mellitus	11	4	7	0.3377
Hypertension	10	2	8	0.0455
Cardiac disease	1	0	1	1.0000
Poliomyelitis	2	2	0	0.4751
Mental disorder	2	1	1	1.0000
Hypophrenia	2	1	1	1.0000
**Hospitalization data**			
Time from admission to MRSA detected, median days (range)	5 (1–54)	5.5 (1–54)	4.5 (1–37)	0.1235
Hospital admission(s) during past year	40	21	19	0.6831
Transfer from another hospital	17	10	7	0.4245
Blood-derivates transfusion	10	6	4	0.5050
**Burn wound characteristics**			
Time from burn to admission, median days (range)	1 (0.5 h−90 days)	10 h (1 h−90 days)	2 (0.5 h−90 days)	0.8601
Time from burn to MRSA detected, median days (range)	13 (1–98)	11 (1–98)	14 (1–91)	0.8894
Percentage of body surface involved, median (range)	6 (0.1–95)	6 (0.1–95)	4 (0.2–95)	0.8651
Burn by direct exposure to flame	45	29	16	0.0090
Scalding	50	19	31	0.0164
Electrical injury	5	2	3	1.0000
Third degree burn	67	37	30	0.1366
Head involvement	41	21	20	0.8389
Upper limbs involvement	49	28	21	0.1614
Trunk involvement	52	28	24	0.4233
Perineal involvement	15	4	11	0.0499
Lower limbs involvement	64	31	33	0.6769
Procedure of Skin grafting	73	36	37	0.8218
**Antimicrobial use**			
**External use**			
Silver sulfadiazine/Sulfamylon	54	16	38	< 0.0001
Rifampin	2	1	1	1.0000
Mupirocin	10	2	8	0.0455
**Non-external use**			
Clindamycin	13	11	2	0.0074
Teicoplanin	4	4	0	0.1258
Imipenem	9	4	5	1.0000
Cefperazone/Sulbactam	5	5	0	0.0665
Vancomycin	19	11	8	0.4444
Ceftazidime	14	6	8	0.5644
Cefuroxime	25	5	25	< 0.0001
Meropenem	5	1	4	0.3588
Linezolid	4	2	2	1.0000
Colistin	7	2	5	0.4331
Amikacin	3	1	2	1.0000

### Antimicrobial resistance

All MRSA isolates in this study have been *mecA*-confirmed by PCR. No isolate was found resistant to quinupristin/dalfopristin, linezolid, and vancomycin. In addition to cefoxitin, benzylpenicillin, and oxacillin, all the MRSA isolates were resistant to tigecycline as well. The resistance rates of MRSA isolates to gentamicin, ciprofloxacin, levofloxacin, moxifloxacin, tetracycline, and rifampin were significantly higher in 2013–2017 (*P* < 0.05), as shown in Table 2. However, inducible clindamycin resistance was higher in 2018–2022 (*P* = 0.0166).

**Table 2:** The antibiotic resistance rates of MRSA isolated from burn wound infection from 2013 to 2022.

**Antibiotics**	**Resistance rate (%)**			
	**Total (*****n*** = **100)**	**2013–2017 (*****n*** = **50)**	**2018–2022 (*****n*** = **50)**	***P*** **-value**
Cefoxitin Screen	100	100	100	–
Benzylpenicillin	100	100	100	–
Oxacillin	100	100	100	–
Gentamicin	74	94	54	< 0.0001
Ciprofloxacin	82	98	66	< 0.0001
Levofloxacin	82	98	66	< 0.0001
Moxifloxacin	82	98	66	< 0.0001
Inducible Clindamycin Resistance	17	8	26	0.0166
Erythromycin	89	94	84	0.1100
Clindamycin	89	94	84	0.1100
Quinupristin/Dalfopristin	0	0	0	–
Linezolid	0	0	0	–
Vancomycin	0	0	0	–
Tetracycline	74	94	54	< 0.0001
Tigecycline	100	100	100	–
Rifampin	27	38	16	0.0132
Trimethoprim/Sulfamethoxazole	46	54	38	0.1085

### Toxin genes

The *seg* was found most frequently (90%) followed by *sea* (70%) and *eta* (64%), as shown in Table 3. The *eta* and *sea* were found more frequently among the MRSA isolates in 2013–2017 (*P* < 0.0001 and *P* = 0.0001, respectively). However, *seb* was observed more frequently among the MRSA isolates in 2018–2022 (*P* = 0.0196). The toxin gene *etb* has not been discovered in this study.

**Table 3:** Prevalence of toxin genes among MRSA isolated from burn wound infection from 2013 to 2022.

**Toxin genes**	**Positive rate (%)**			
	**Total (*****n*** = **100)**	**2013–2017 (*****n*** = **50)**	**2018–2022 (*****n*** = **50)**	***P*** **-value**
*lukS/F-PV*	3	0	6	0.2410
*tst*	3	6	0	0.2410
*eta*	64	94	34	< 0.0001
*etb*	0	0	0	–
*sea*	70	92	48	< 0.0001
*seb*	10	2	18	0.0077
*sec*	5	6	4	1.0000
*sed*	2	0	4	0.4751
*see*	3	2	4	1.0000
*seg*	6	4	8	0.6737
*seh*	90	86	94	0.1824
*sei*	7	6	8	1.0000
*sej*	2	2	2	–

### Molecular types

In total, 11 sequence types (STs) and 7 clonal complexes (CCs) were identified, as shown in Table 4. CC8 (74%) and ST239 (70%) were the most common CC and ST, respectively, and CC8 and ST239 came from the same burn units. SCC*mec* III (72%) was the most frequently found SCC*mec* type, followed by SCC*mec* I (15%), SCC*mec* V (11%), SCC*mec* III (1%), and SCC*mec* NT (1%). One MRSA isolate (ST2315-SCC*mec*NT-t11687) was unable to be SCC*mec*-typed by PCR. Whole-genome sequencing was performed on this isolate to obtain more information about SCC*mec* elements, but it still cannot by SCC*mec* typed for lack of *ccr* gene. The sequence data have been deposited in GenBank under BioProject ID PRJNA1024061 (http://www.ncbi.nlm.nih.gov/bioproject/1024061). As to *spa* types, t037 (41%) was the most common type, followed by t030 (11%) and t459 (7%). The *agrI* (91%) was the most frequent *agr* group, followed by *agrII* (6%) and agr*III* (3%). The distribution of MRSA clones in 2013–2017 and 2018–2022 is presented in Table 5. ST239-III was the most common clone accounting for 70%, and ST239-III was more found in 2013–2017 (*P* < 0.0001); however, ST59-I was more found in 2018–2022 (*P* = 0.0099). In total, 4 and 14 MRSA clones were found in 2013–2017 and 2018–2022, respectively, and the diversity of MRSA clones in 2018–2022 was much greater than that in 2013–2017 (*P* = 0.0004).

**Table 4:** Molecular characteristics of MRSA isolated from burn wound infection from 2013 to 2022.

**CC (*n*)**	**ST (*n*)**	**SCC*mec* (*n*)**	***spa* type (*n*)**	**Toxin genes (*n*)**
8 (74)	239 (70)	III (70)	t037 (40), t030 (11), t459 (7), t421 (5), t632 (5), t932 (1), New1 (1)	*sea* (60), *sed* (1), *see* (1), *seh* (63), *sej* (1), *eta* (52)
	630 (3)	V (3)	t4549 (2), New2 (1)	*seh* (1), *eta* (1),
	8 (1)	V (1)	t9101 (1)	*sed* (1), *sej* (1)
59 (9)	59 (9)	I (8)	t163 (3), t437 (2), t172 (1), t3485 (1), t3527 (1)	*lukS/F-PV* (2), *sea* (5), *seb* (6), *seh* (8), *eta* (1)
	V (1)	t437 (1)	*lukS/F-PV* (1), *seb* (1), *seh* (1), *eta* (1)
5 (7)	5 (6)	I (3)	t311 (2), t002 (1)	*tst* (2), *sea* (2), *seb* (1), *sec* (2), *seg* (2), *seh* (3), *sei* (3), *eta* (2)
		III (2)	t002 (1), t037 (1)	*tst* (1), *seb* (1), *sec* (1), *seg* (1), *seh* (2), *sei* (1), *eta* (2)
		II (1)	t002 (1)	*seb* (1), *see* (1), *seg* (1), *seh* (1), *sei* (1), *eta* (1)
	6 (1)	I (1)	t034 (1)	*sea* (1), *seh* (1)
398 (6)	398 (6)	V (6)	t034 (5), t1451 (1)	*see* (1), *seh* (6), *eta* (3)
1 (2)	1 (1)	I (1)	t127 (1)	*sea* (1), *sec* (1), *seh* (1), *eta* (1)
	2315 (1)	NT (1)	t11687 (1)	*sea* (1), *sec* (1), *seg* (1), *seh* (1), *sei* (1)
45 (1)	3351 (1)	I (1)	t116 (1)	*seg* (1), *seh* (1), *sei* (1)
88 (1)	88 (1)	I (1)	t3622 (1)	*seh* (1)

**Table 5:** Distribution of MRSA clones from burn wound infection from 2013 to 2022.

**Clone**	**Total (*n*)**	**2013–2017 (*n*)**	**2018–2022 (*n*)**	***P*-value**
ST239-III	70	46	24	< 0.0001
ST630-V	3	0	3	0.2410
ST8-V	1	0	1	1.0000
ST59-I	8	0	8	0.0099
ST59-V	1	0	1	1.0000
ST5-I	3	2	1	1.0000
ST5-III	2	1	1	1.0000
ST5-II	1	0	1	1.0000
ST6-I	1	0	1	1.0000
ST398-V	6	1	5	0.2065
ST1-I	1	0	1	1.0000
ST2315-NT	1	0	1	1.0000
ST3351-I	1	0	1	1.0000
ST88-I	1	0	1	1.0000

## Discussion

Burn is one of the most common and devastating forms of trauma, and patients with severe thermal injuries require immediate specialized medical care to minimize morbidity and mortality (Church et al., 2006). However, burn patients with infections presented more than twice the mortality rate of uninfected burn patients. It has been reported that 42–65% of deaths in burn patients can be attributed to infections over the past decades (D'Abbondanza and Shahrokhi, 2021). The bacteria responsible for infection appear in chronological order, changing with the time since the initial burn happened. So far, one of the most common pathogens involved in burn wound infections remains *S. aureus* (D'Abbondanza and Shahrokhi, 2021). The prevalence of multidrug-resistant (MDR) bacteria in burn units may lead to a vicious cycle of increasing antibiotic resistance by selecting antibiotics empirically that target MDR bacteria (Lachiewicz et al., 2017). Therefore, MRSA deserves more attention as one of the most common pathogens in burn infections. In addition to the loss of skin barrier function, burns may lead to a certain degree of immune suppression, which, in turn, makes the pathogen MRSA more aggressive (Kaiser et al., 2011).

Our study is a 10-year retrospective of MRSA from burn wound infections from 2013 to 2022, and we split it into two groups by year as 2013–2017 and 2018–2022 to find out how MRSA might change over time. As shown in Table 1, in the last 5 years in 2018–2022, the incidence of burn by direct exposure to flame was lower and instead the incidence of burn by scalding was higher. More notably, topical antibiotics such as silver sulfadiazine/sulfamylon and mupirocin were more frequently used in 2018–2022, which can prevent infections in burn patients. The microbial load on the open burn wounds and the risk of infections could be greatly reduced by the wide application of effective topical antimicrobials. A series of studies have demonstrated the role of topical antimicrobials in reducing morbidity and mortality among burn patients with severe burn injuries (partial-thickness or full-thickness skin involvement), especially before early excision (Church et al., 2006).

Several studies have shown that partial antibiotic resistance rates of *S. aureus* and MRSA have declined in recent years (Murray et al., 2009; Jiang et al., 2018; Mahmoudi et al., 2019). In our study, the resistance rates of MRSA to gentamicin, ciprofloxacin, tetracycline, rifampin, and other series antibiotics from 2018 to 2022 were significantly lower than that in 2013–2017. However, inducible clindamycin resistance was higher in 2013–2017. It is still unclear what accounts for this trend of MRSA resistance, or if it is simply due to chance. One research studied vancomycin susceptibility trends of MRSA isolated from burn wounds and found that the proportion of MRSA isolates exhibiting higher vancomycin MICs increased significantly (Zorgani et al., 2015). In our study, the 75% vancomycin MIC value of MRSA isolates was 2 μg/ml; the 21% vancomycin MIC value was 1 μg/ml, and only 4% vancomycin MIC value was ≤0.5 μg/ml. Those high MIC values indicate that vancomycin heteroresistance might emerge, although relevant information was not available. However, there is an urgent need to implement infection control measures to prevent the spread of MRSA, especially in burn patients.

In addition to the high positive rates of enterotoxin genes such as *seh* (90%) and *sea* (70%), exfoliative toxin A (ETA) gene *eta* was also frequently detected accounting for 64%, which was much higher than the *S. aureus* isolates we studied before from bloodstream infections, skin and soft tissue infections, and colonization (Gu et al., 2015, 2016, 2020; Zhang et al., 2015; He et al., 2021). Staphylococcal exfoliative toxins are responsible for staphylococcal scalded skin syndrome, which is characterized by dehydration, detachment of superficial skin layers, and secondary infections (Ahmad-Mansour et al., 2021). The prevalence of ETA in MRSA and methicillin-susceptible (MSSA) strains does not differ considerably as reported; nevertheless, 4% of MSSA strains carry the *eta* or *etb* gene, while approximately 10% of MRSA strains possess the *eta* gene, according to a recent study (Ahmad-Mansour et al., 2021). However, resistant strains such as MRSA with *eta*-positive may pose a problem nowadays or in future, especially among burn patients.

CC8 (74%) was found as the dominant clonal complex in MRSA burn wound infection among burn patients, and ST239-III was the most common clone with 70%. CC8 or ST239-III is a predominant healthcare-associated MRSA (HA-MRSA) clone across the world including America, Europe, Africa, Middle East, and Asia (Lakhundi and Zhang, 2018). As to burn infections, a study conducted at Jiangxi Burn Center in China reported that SCC*mec*III-CC239-t030 was the most common clone (Chen et al., 2018). ST239-SCC*mec* III/t037 has emerged as the major MRSA clone in burn patients in Iran as reported in 2017 (Goudarzi et al., 2017). It is interesting that ST239-III was less found in recent 5 years in 2018–2022 (46 vs. 24, *P* < 0.0001), and ST59-I was more found in 2018–2022 (0 vs. 8, *P* = 0.0099) in our study. The diversity of MRSA clones from 2018 to 2022 was obviously greater than that in 2013–2017 (4 vs. 14, *P* = 0.0004), as shown in Table 5. It might suggest that besides the predominant clone ST239-III, other MRSA clones should also be watched and monitored among burn patients. In this study, livestock-associated MRSA (LA-MRSA) clone CC398-V occurred in 2017, 2018, 2020, 2021, and 2022 has emerged as a cause of hospital outbreaks, and more discoveries in recent years might indicate its greater ability to spread among patients in hospital along with time. ST398 has been reported as one of the predominant MRSA clones with ST239 in hospitals in India (Patil et al., 2023), and MRSA CC398 has been described in human colonization and infections over these years including our previous studies (Ballhausen et al., 2017; Gu et al., 2020; He et al., 2021; Silva et al., 2022).

MRSA colonization upon admission and during hospitalization among burn patients cannot be ignored, and it could result in multiclonal MRSA outbreaks in the burn units if decolonization protocols are not implemented (Kalligeros et al., 2019; Kim et al., 2019). The implementation of universal decolonization with intranasal mupirocin was effective as reported, and the prevalence of HA-MRSA in burn centers was significantly decreased after the application of the decolonization protocol (Johnson et al., 2016; Kim et al., 2019). Burn patients colonized or infected with MRSA are more likely to be a main reservoir transfer to others; a comprehensive concept to control the spread of all multidrug-resistant pathogens including MRSA is deeply needed in burn units, perhaps temporarily shut down, supplemented by intensive cleaning, are effective measures to stop the transmission events (Baier et al., 2018).

## Data availability statement

The original contributions presented in the study are publicly available. This data can be found at: https://www.ncbi.nlm.nih.gov/bioproject; PRJNA1024061.

## Author contributions

FG: Conceptualization, Data curation, Funding acquisition, Methodology, Writing – original draft, Writing – review & editing. WH: Data curation, Methodology, Writing – original draft. DZ: Investigation, Software, Writing – original draft. PY: Data curation, Writing – review & editing. JS: Conceptualization, Supervision, Writing – review & editing. LH: Formal analysis, Project administration, Writing – review & editing.
